# Local and global convolutional transformer-based motor imagery EEG classification

**DOI:** 10.3389/fnins.2023.1219988

**Published:** 2023-08-17

**Authors:** Jiayang Zhang, Kang Li, Banghua Yang, Xiaofei Han

**Affiliations:** ^1^School of Electrical Engineering, University of Leeds, Leeds, United Kingdom; ^2^School of Mechatronic Engineering and Automation, Shanghai University, Shanghai, China

**Keywords:** brain-computer interface, motor imagery, transformer, attention mechanism, Convolutional Neural Network

## Abstract

Transformer, a deep learning model with the self-attention mechanism, combined with the convolution neural network (CNN) has been successfully applied for decoding electroencephalogram (EEG) signals in Motor Imagery (MI) Brain-Computer Interface (BCI). However, the extremely non-linear, nonstationary characteristics of the EEG signals limits the effectiveness and efficiency of the deep learning methods. In addition, the variety of subjects and the experimental sessions impact the model adaptability. In this study, we propose a local and global convolutional transformer-based approach for MI-EEG classification. The local transformer encoder is combined to dynamically extract temporal features and make up for the shortcomings of the CNN model. The spatial features from all channels and the difference in hemispheres are obtained to improve the robustness of the model. To acquire adequate temporal-spatial feature representations, we combine the global transformer encoder and Densely Connected Network to improve the information flow and reuse. To validate the performance of the proposed model, three scenarios including within-session, cross-session and two-session are designed. In the experiments, the proposed method achieves up to 1.46%, 7.49% and 7.46% accuracy improvement respectively in the three scenarios for the public Korean dataset compared with current state-of-the-art models. For the BCI competition IV 2a dataset, the proposed model also achieves a 2.12% and 2.21% improvement for the cross-session and two-session scenarios respectively. The results confirm that the proposed approach can effectively extract much richer set of MI features from the EEG signals and improve the performance in the BCI applications.

## 1. Introduction

The brain-computer interface (BCI), as a promising tool for stroke rehabilitation and other biomedical applications, enables people to interact with external devices by decoding the electroencephalogram (EEG) signals generated by various brain activities (Mane et al., 2020a). EEG is widely used in the clinical and neuroscience domain, especially in BCI systems because of its excellent properties of noninvasiveness and portability (Zhang and Li, 2022). Motor imagery (MI), the mental rehearsal of physical movement task (Decety and Ingvar, 1990), is commonly used to allow disabled people to self-regulate EEG signals through active modulation rather than external stimulus and assistance. When a person is imagining a certain motor behavior, the related motor cortex generates the corresponding neuron responses which help improve motor functional recovery by reducing the gap induced by brain disorders between motor intention and sensory feedback of motor movements (Craik et al., 2019).

However, the non-stationary, low signal-to-noise ratio and non-linear characteristics make it difficult to decode MI-EEG signals. Traditional machine-learning approaches mainly focus on analyzing the spatial information from MI-EEG signals. The representative method called the Common Spatial Pattern (CSP) is valid in building optimal spatial filters to distinguish binary MI tasks (Pfurtscheller and Neuper, 2001). The improved variants of the CSP method such as Common Spatio-Spectral Pattern (CSSP) (Lemm et al., 2005), Sub-band Common Spatial Pattern (SBCSP) (Novi et al., 2007) and Filter Bank Common Spatial Pattern (FBCSP) (Ang et al., 2008) have also been implemented successfully in MI classification. Further, Alexandre et al. proposed the minimum distance to Riemannian mean (MDM) and tangent space mapping (TSM) (Barachant et al., 2011) for the EEG classification by using the topology of the manifold of symmetric and positive definite (SPD) matrices. Conventional classifiers such as Linear Discriminant Analysis (LDA) (Chen et al., 2014) and Support Vector Machines (SVM) (Li et al., 2013) are common methods used in MI-BCI. Although many methods have achieved impressive results, the unfitting and inefficient combination of feature extraction methods and classifiers limits the model's accuracy, robustness, and adaptability performance.

As an end-to-end signal processing method, Deep Learning (DL) has been successfully applied in extracting and analyzing abstract information from MI-EEG signals in recent years (Lotte et al., 2007; Zancanaro et al., 2023). For example, Schirrmeister et al. proposed several models based on the Convolutional Neural Networks (CNNs) according to the principle of FBCSP (Schirrmeister et al., 2017). The two first layers used in the model capture temporal and spatial features which greatly influence subsequent studies. Lawhern et al. (2018) replaced the common CNN layer with the separable one to reduce the calculated dimensions. The hyper-parameters were also adopted according to the sampling rate and brain rhythms. To further improve the capabilities of the DL model based on CNNs, Dai et al. (2020) and Wu et al. (2019) used multiscale CNN filters to capture the features in different fields of view. Huang et al. adopted the SPD matrices as inputs to capture the spatial patterns of EEG signals (Huang and Van Gool, 2017). Syed et al. fused the results from four parallel CNN structures with multi-depth for fusing shallow and deep layers to learn the relevant MI information at different levels (Amin et al., 2019). Mane et al. (2020b) proposed a multi-view CNN namely Filter-Bank Convolutional Network(FBCNet) to encode spectro-spatial discriminative information from MI-EEG with various spectral filtering. Zhang et al. (2019) proposed a hybrid architecture based on the CNN and Long Short-term Memory (LSTM) for processing time series signals. However, the characteristics of hard inductive bias in the CNNs and LSTM are overly restrictive, limiting the potential performance (Chen et al., 2018). The locality of CNNs impairs the ability to extract features from long-range signals like MI-EEG. The huge serial computing consumption and restricted sequence length in LSTM bring the challenge in decoding MI-EEG signals.

In recent years, the DL model based on the transformer (Vaswani et al., 2017) with a multi-head attention mechanism has been successfully applied in the Computer Vision (CV) and Natural Language Processing (NLP) domain. Compared with the CNN or LSTM structure, the transformer has soft inductive bias (d'Ascoli et al., 2021) increasing the upper limit of model performance. The Self-Attention (SA) structure and parallel computing mode allow the transfer extract global information without multiple convolutions and pooling calculations. In the BCI field, the transformer is adopted to handle signals in the applications such as person identification (Du et al., 2022), emotion recognition (Li et al., 2022), visual stimulus classification (Bagchi and Bathula, 2022) and signal denoising (Pu et al., 2022). For MI-EEG decoding, Ma et al. (Ma et al., 2022) proposed a hybrid CNN-Transformer model to weigh spatial features and frequency signals by employing the attention mechanism. However, the model uses the CSP features as inputs which loses the advantage of the end-to-end process in the DL model. Song et al. (2023) also proposed a hybrid model with six transformer encoders after extracting features from MI-EEG by CNN layers. The model performs well in the hold-out tests, but the huge computational costs caused by encoders limit the actual use. Tao et al. (2021) employed the gating mechanism on the transformer to improve the model performance, but missed the extraction of EEG spatial information. Xie et al. (2022) designed five hybrid models with different layers in the CNN and transformer. This study adapted the model in the cross-subject scenario with much more training data than small data scenarios like within-subject and within-session applications, limiting the model robustness. Besides that, the shortfall of these studies is that they only extract the spatial features from the fusion of all channels, neglecting the possible information learned from the differences between the hemispheres.

To address the above issues, a novel approach with the local and global transformer combing with CNNs for MI-EEG classification is proposed in this study. First, we adopt the local transformer and 1-dimension CNN filter with the same kernel size to extract temporal features from each channel. Although the respective fields from the local transformer and CNN are the same in the beginning, the different mechanisms allow the model to learn a comprehensive set of useful and subtle features from multi-views. The local transformer also avoids the overfitting problem compared with the global transformer which extracts more subtle features from raw EEG signals in the first layer. Then, two parallel branches use different depthwise CNN to extract and fuse different spatial information. One branch focuses on all channels in the motor cortex and the other one extracts the features of channels from the left and right motor regions respectively. Next, for better mining the temporal-spatial features, we use the Densely Connected CNN (DenseNet) (Huang et al., 2017) on both CNN and global transformer layers by connecting each layer to every other in a feed-forward way. The short path helps the information reuse and flow which improve the model adaptability and robustness. Finally, the proposed model is validated and compared with other baseline models in different scenarios including within-session and cross-session to verify its performance.

## 2. Materials and methods

In this section, we first introduce the dataset and preprocessing approach used in the experiment briefly. The different scenarios are given in detail. Then, we present the proposed model including the mechanism, structure and hyper-parameters.

### 2.1. Dataset and preprocessing

We used the Korea University dataset (Lee et al., 2019) and the BCI Competition IV 2a (Tangermann et al., 2012) dataset to evaluate the proposed model performance on the two-class and four-class MI tasks classification.

(1) Korea University (KU) Dataset: We used the Korea University Dataset containing 54 subjects with binary MI tasks of the left hand and right hand. Two sessions were conducted on different days in the dataset, each with 200 trials for every subject. The MI-EEG signals were collected by 62 Ag/AgCl electrodes with impedances of less than 10 *kΩ*. To better decode MI information, 20 electrodes in the motor cortex region were selected (C-z/1/2/3/4/5/6, CP-z/1/2/3/4/5/6, FC-1/2/3/4/5/6) according to previous studies (Kwon et al., 2019; Mane et al., 2020b; Ju and Guan, 2022). The sampling rate was 1,000 Hz and we downsampled to 250 Hz.

(2) BCI Competition IV 2a (BCIC-IV-2a) Dataset: The BCIC-IV-2a consists of recordings from nine healthy subjects performing four different motor imagery tasks: left-hand, right-hand, both-foot, and tongue. The signals were acquired using 22 EEG electrodes with a sampling frequency of 250 Hz and were bandpass filtered between 0.5 Hz and 100 Hz, as well as notch filtered at 50 Hz. Two sessions were recorded on different days for each subject, with each session comprising 288 trials. The dataset only has 22 channels so that we feed all channel signals into the proposed model.

The most common frequency band used in the MI-EEG field is α rhythm (Jasper and Andrews, 1938) which is about 10 Hz and β rhythm which is around 20 Hz (Jasper and Penfield, 1949). The filter bands that include useful spectral MI information vary from person to person (Novi et al., 2007). Therefore, some studies (Kwon et al., 2019; Mane et al., 2020b; Ma et al., 2022) divided the raw MI signals into several bands with a 4 Hz length ranging from 4 to 40 Hz by spectral filters. Considering the extra calculations caused by multi-inputs, we only feed three inputs including the raw signals and two filtered bands based on α (7–12 Hz) and β (13–32 Hz) rhythms. Each trial has 4 s with 1,000 samples in total. We employed the Z-score normalization to handle the signals, as shown:


(1) $$\begin{array}{l} Z=\frac{x-\mu }{\sigma } \end{array}$$

where *x* was the raw data of each channel. μ was the mean value of *x* and σ represents the standard deviation.

### 2.2. Scenarios description

**Figure 1:**
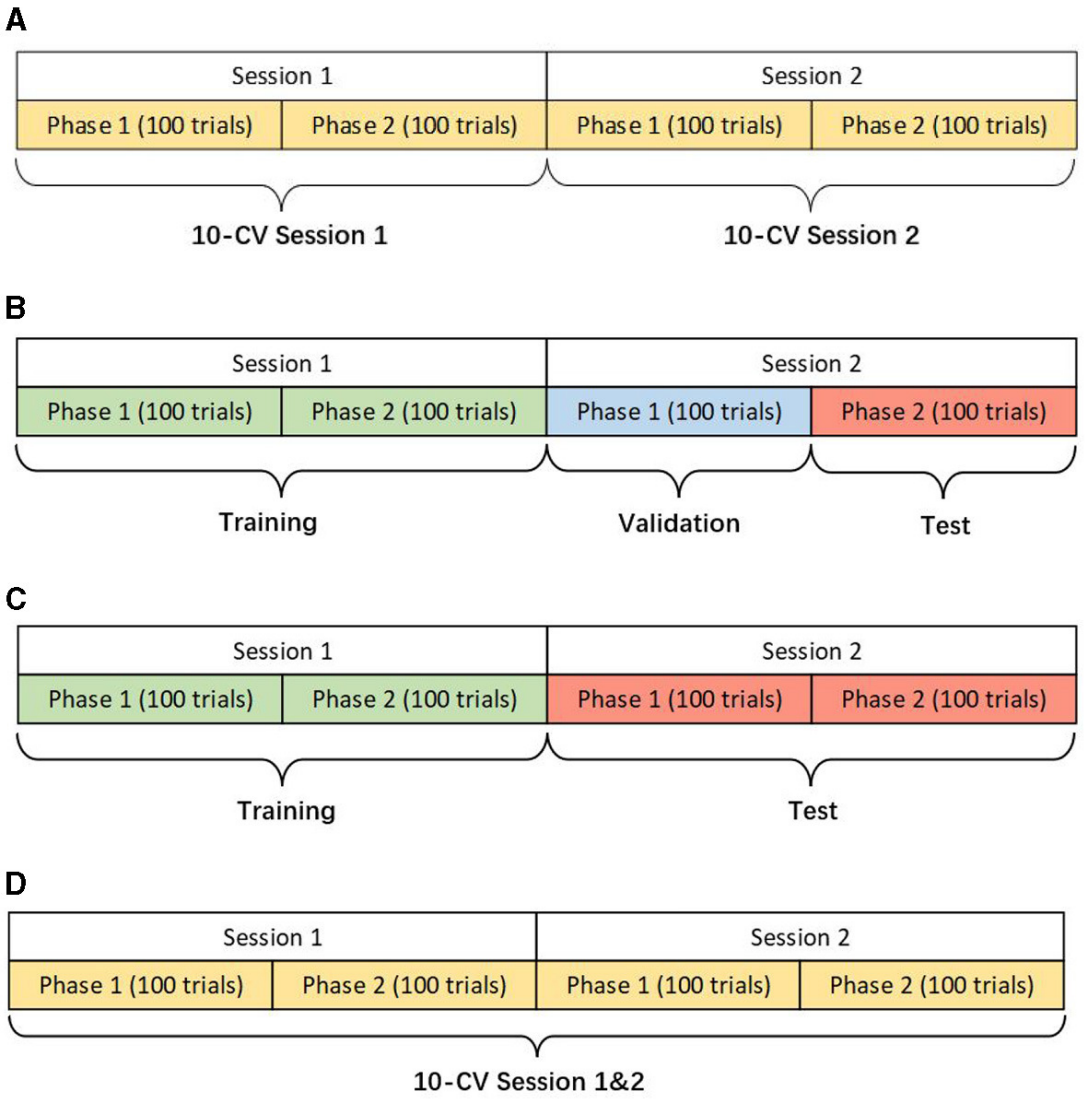
Descriptions of different scenarios (KU dataset). **(A)** Within-session scenario. **(B)** Cross-session scenario case 1. **(C)** Cross-session scenario case 2. **(D)** Two-session scenario.

We design three scenarios of the within-subject analysis using data from the same subject for training, validation, and testing (Figure 1). Different scenarios help verify the models' adaptability and robustness for actual applications. The details of different scenarios are described as follows:

1. Within-Session Scenario: This scenario only uses one session with 200 trials for 10-fold cross-validation (CV). Although the training data is limited, within-session ensures the stability of the data distribution as far as possible.
2. Cross-Session Scenario: The first session is used for training and the second one for testing. Two cases are presented in this scenario considering the different applications in reality. The first namely the hold-out scenario uses the part of the data in session two for validation and the rest for the test. The other case only uses the whole data in session two for the test, ensuring no data participates in validation at the modeling stage. In either case, the data from session two will not be used in training. Due to the circumstance that two sessions were conducted on different days, the drift of statistical distributions brings the challenge for classification.
3. Two-Session Scenario: Two sessions of one subject are grouped for a 10-fold CV to show the performance of the models in big data.

In the BCIC-IV-2a dataset, each phase contains 144 trials because there are 288 trials for each session while one phase in the KU dataset has only 100 trials.

### 2.3. The proposed model

#### 2.3.1. Architecture

**Figure 2:**
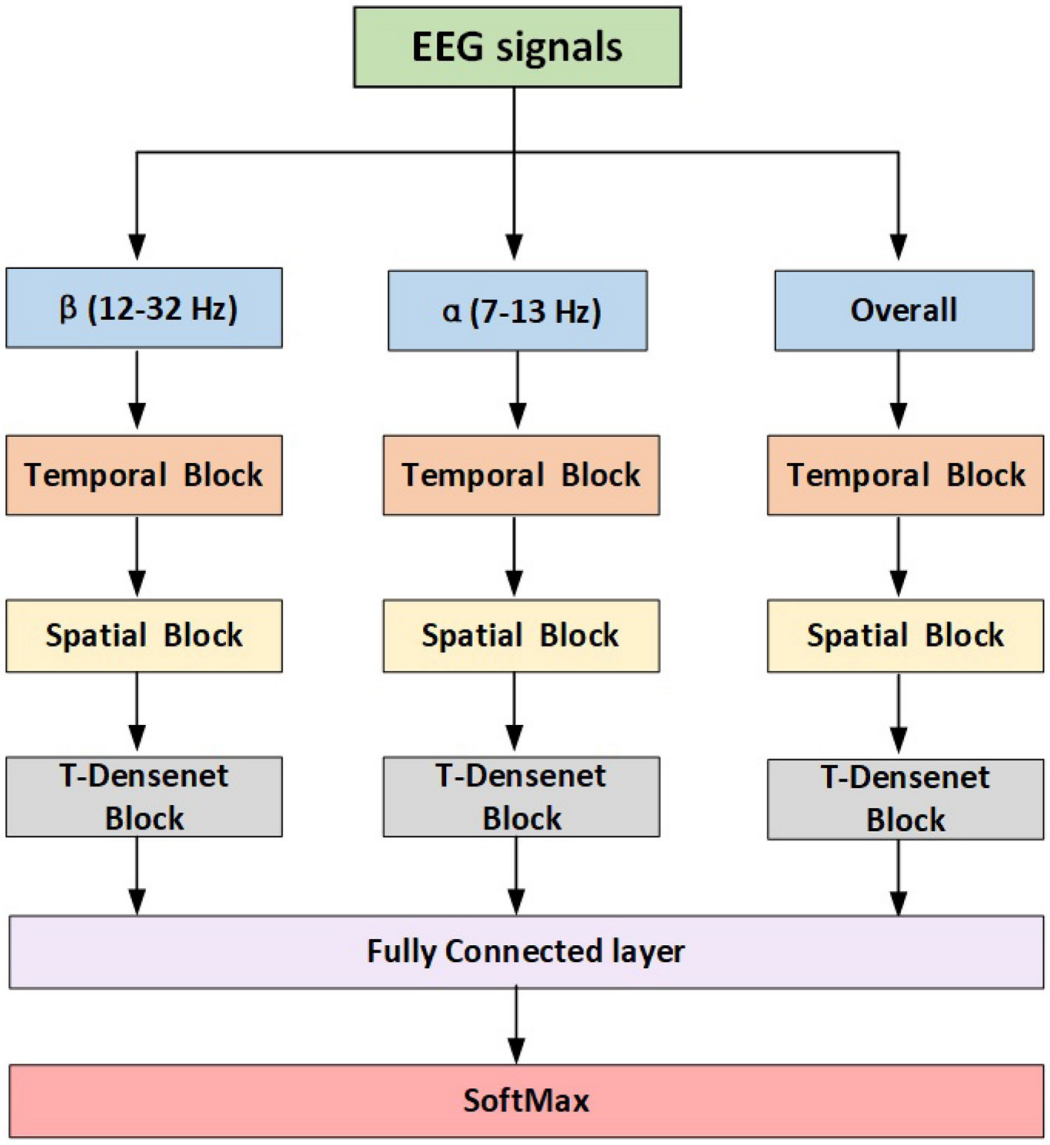
The proposed model structure.

The proposed model has three branches which were fed from filtered data and concentrated by a fully connected layer for fusing features from multi-bands. Each branch has the same structure consisting of the temporal block, spatial block and transformer-based densenet block (T-Densenet Block) (Figure 2).

#### 2.3.2. Temporal block

Considering that the MI-EEG signals are time series, the previous studies (Schirrmeister et al., 2017; Lawhern et al., 2018; Mane et al., 2020b) preferred using a 1-D CNN filter to extract the temporal feature which is one of the most distinguished MI information. CNN filter has a strong inductive bias of weight sharing (Simoncelli and Olshausen, 2001). Such a characteristic reduces a huge amount of computation and makes a model more parameter-efficient, but it ignores the dynamic relationship among the input data in a kernel with the filter sliding because the weights learned by the CNN are fixed after training.

##### 2.3.2.1. Self-attention

**Figure 3:**
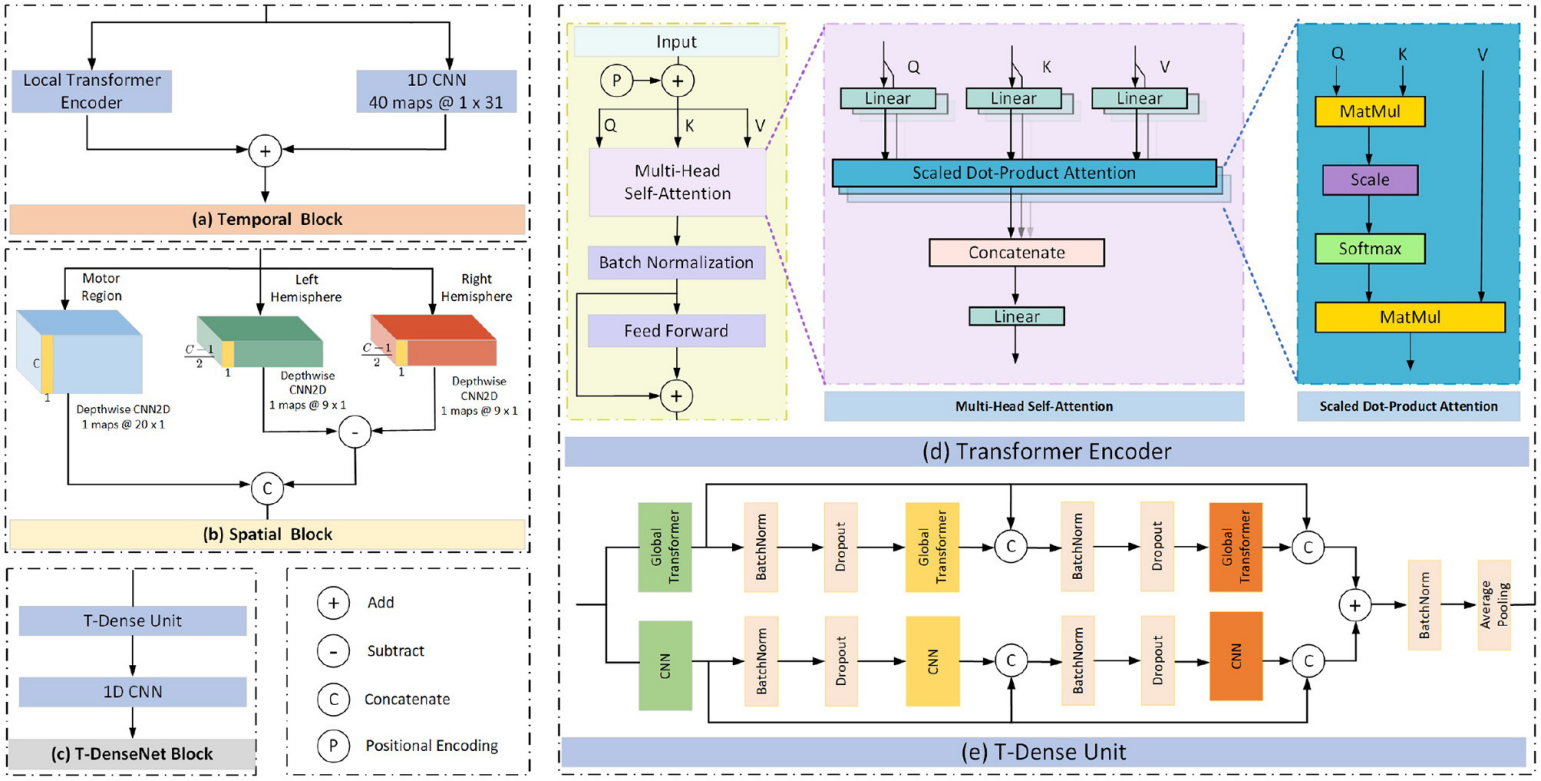
The proposed model structure.

The self-attention mechanism focuses more on the correlation between each value in the kernel and all other values. First, the transformer encoder divides the input into three representations namely Queries (Q), Keys (K) and Values (V) by the linear dense layers. Then the specific attention “Scaled Dot-Product Attention” (shown in Figure 3d) computed the dot products of the queries with all keys. The results were divided by $\sqrt{d_{k}}$ and ended with a softmax function to obtain the weights on the values. The formula is:


(2) $$\begin{array}{l} Attention\;\left(Q,K,V\right)=softmax\;\left(\frac{QK^{T}}{\sqrt{\begin{array}{l} \begin{array}{l} d_{k} \end{array} \end{array}}}\right)\;V \end{array}$$

where *d*_*k*_ was the dimension of keys. To better jointly learn the information from different representation subspaces at different positions (Vaswani et al., 2017), the scaled dot-product attention was embedded in the structure of the “Multi-head Self Attention” (Figure 3d):


(3) $$\begin{array}{l} MultiHead\left(Q,K,V\right)=Concat\left(head_{1},...,head_{h}\right)W^{O}\\ head_{i}=Attention\left(Q,K,V\right) \end{array}$$

where $W^{O}\in R^{hd_{v}\times d_{model}}$, *hd*_*v*_ resprents the dimension of values and *d*_*model*_ is the dimension of the outputs. We employ *h* = 2 parallel attention layers in the proposed model. From Equations (2) and (3), the calculation of the output is determined by a weighted total of the values, and the weight for each value is determined by a function that assesses the compatibility between the query and its corresponding key. Therefore, the weights are dynamic rather than fixed like CNN filters.

##### 2.3.2.2. Local transformer encoder

**Figure 4:**
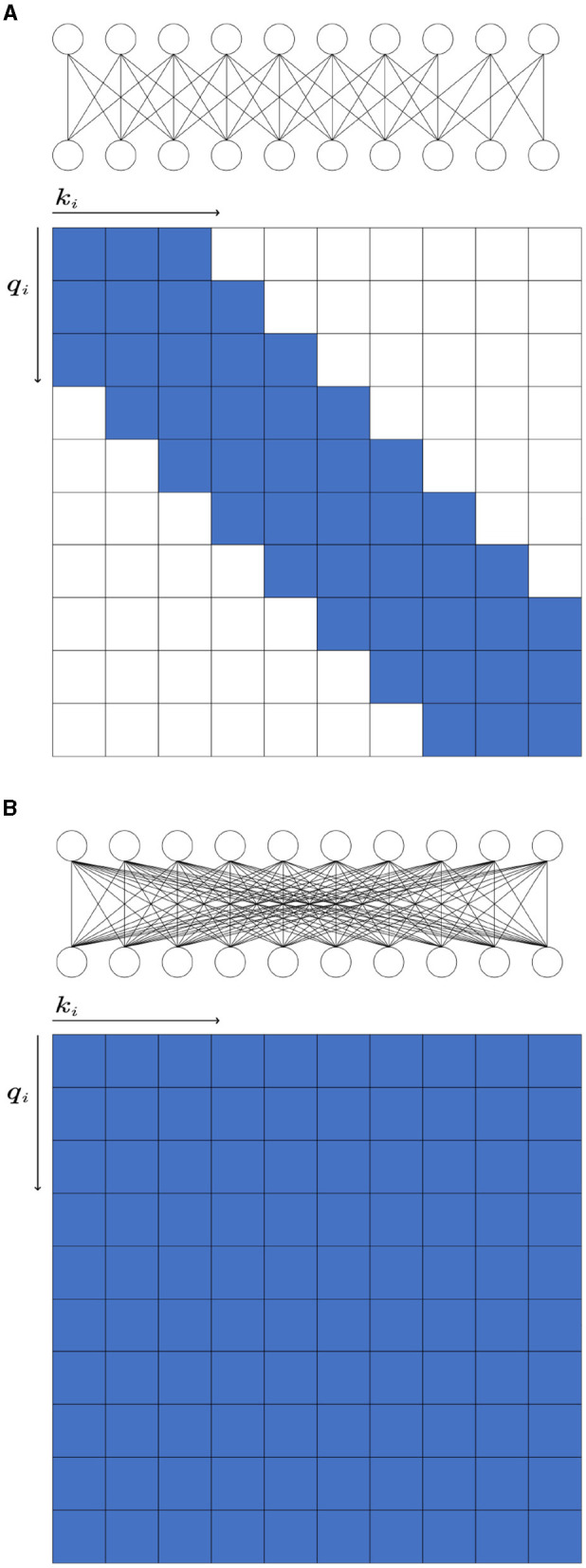
Attention patterns in the transformer. The blue squares represent corresponding attention scores are calculated and the blank ones mean the attention score is discarded. **(A)** Local pattern. **(B)** Global pattern.

In this work, to take full advantage of the characteristics of the modes of CNN and transformer, we add the outputs from the local transformer encoder and those from the CNN filter together as the final temporal features (Figure 3a). Compared with the global transformer that obtains the attention score of a query based on all keys (Figure 4B), the local transformer encoder reduces the number of keys to ensure that the queries are multiplied by the limited keys every time (Figure 4A). Such a mode improves the temporal feature decoding by increasing the locality. Although the local mode cannot learn the global features, it selects local subtle features which otherwise are largely ignored in the global mode. And it can further overcome the overfitting and underfitting problems for long raw EEG signals.

##### 2.3.2.3. Positional encoding

Considering that the MI-EEG signals are the sequence that has the order, the position information is injected by the sum of the Positional Encoding (PE) value and the raw signals. According to the successful PE application in the MI-EEG field (Xie et al., 2022), we used sine and cosine functions to represent the position as follows:


(4) $$\begin{array}{l} PE_{\left(pos,2i\right)}=\sin\left(\frac{pos}{10000^{2i/d}}\right) \end{array}$$

(5) $$\begin{array}{l} PE_{\left(pos,2i+1\right)}=\cos\left(\frac{pos}{10000^{2i/d}}\right) \end{array}$$

where *pos* means the position and *i* is the dimension. *d* represents the dimension of the inputs.

#### 2.3.3. Spatial block

Previous research has already demonstrated the feasibility of using the brain hemisphere to control both the left and right hands, but the degree of control for each hand differs due to lateralization (Müller et al., 1998; Martin et al., 2016). Therefore, the spatial feature differences between the two hemispheres may potentially be useful for motor imagery classification. After concatenating the temporal features learned from the CNN and the local transformer encoder, the depthwise CNNs are used to extract spatial information from the EEG channels. The proposed model sends the input into three parallel paths (Figure 3b). The first CNN filters extract the spatial features from all C channels in the motor region. The rest two CNN filters extract features from $\frac{C-1}{2}$ channels in the left hemisphere and the right hemisphere respectively. The extra channel Cz was deleted because it was set in the central position. Then the difference was obtained by subtracting the features of the two hemispheres. Finally, the spatial features based on the channels from the motor region and the difference caused by hemispheres are fed into the next block.

#### 2.3.4. T-Dense block

This block comprises one T-Dense Unit and a 1-D CNN filter, as shown in Figure 3c. The T-Dense Unit (Figure 3e) has two branches with the CNN filters and the global transformers (Figure 4B) respectively. Both branches in the T-dense unit has the similar structure and processing steps as shown in Figure 3e. For instance, in the CNN filter branch, the features of the first CNN filter are concatenated with the ones from the second filter to feed into the third CNN filter. Each subsequent layer of CNN is not only connected to the immediate preceding layer, but also to all other preceding layers, enabling the establishment of shorter paths to help the flow and reuse of the information (Huang et al., 2017). Meanwhile, batch normalization and dropout techniques are applied to address the overfitting issue. In the T-dense unit, the global mode is applied to the transformer to retain its original advantages of extracting the global information using all neurons based on the SA mechanism while CNN filters works differently, as they learn the global information by sliding and pooling layers steps given the limited size of a filter. The branches in the T-Dense Unit are combined to learn a comprehensive set of features which otherwise can not be achieved using a single feature extraction mechanism. After the T-Dense Unit, the 1-D CNN layer is used to reduce the dimension thus to reduce the calculation burden for the subsequent output neurons after three parallel branches.

#### 2.3.5. Training setup

The cross-entropy function is employed as a loss function which evaluates the distance between the probability distribution of the model prediction values *y*_*p*_ and the true labels *y*_*t*_:


(6) $$\begin{array}{l} L\left(y_{p},y_{t}\right)=-\underset{m}{\sum }y_{p,m}\;\log\;y_{t,m}. \end{array}$$

where *m* is the index of *y*. The optimizer is Adam (Kingma and Ba, 2014) and the learning rate is set to 0.0001. The training takes 800 epochs with 32 batches per epoch. The early stopping technology was used to save the best weights. The training step ended after checking if the validation loss value decreased for the last 100 epochs. After reaching the threshold, the model with the best weights produces the classification results of the test fold.

The computer used in this experiment had 15 Intel processors and 80 GB RAM. GTX 3090 GPU with 24 GB memory was used for training and testing MI-EEG signals. Keras based on TensorFlow was used for constructing the proposed model.

## 3. Results

### 3.1. Performance comparison

We evaluate the proposed model and other models in the different scenarios. The average classification accuracies of all subjects of the KU dataset and BCIC-IV-2a with standard deviation (SD) are shown in Tables 1, 2 respectively.

**Table 1:** Comparison of average classification accuracy (%) and standard deviation (SD) for different methods (KU dataset).

	**Within-session**		**Cross-session**		**Two-session**
		**Session1 (SD)**	**Session2 (SD)**	**Case1 (SD)**	**Case2 (SD)**	**Session 1&2 (SD)**
CSP	56.53 (13.10)	58.38 (14.63)	61.70 (16.14)	60.43 (13.98)	55.80 (11.07)
FBCSP	64.41 (16.28)	66.47 (16.53)	59.67 (14.32)	61.57 (14.73)	65.62 (14.75)
MDM	50.47 (8.63)	51.93 (9.79)	52.33 (6.74)	-	-
TSM	54.59 (8.94)	54.97 (9.93)	51.65 (6.11)	-	-
SPDNet	57.88 (8.68)	58.88 (8.68)	60.41 (12.13)	-	-
Shallow ConvNet	67.73 (17.58)	68.47 (17.65)	67.79 (19.16)	66.32 (16.18)	72.74 (15.82)
Deep ConvNet	56.19 (13.71)	57.38 (15.27)	56.59 (15.29)	56.75 (13.03)	62.91 (17.64)
EEGNet	63.37 (17.06)	64.73 (17.97)	65.26 (19.31)	63.28 (15.69)	69.73 (17.05)
FBCNet	74.16 (12.60)	73.81 (13.99)	67.83 (14.34)	-	-
Tensor-CSPNet	74.95 (15.27)	75.92 (13.99)	69.65 (14.97)	-	-
**Proposed model**	**75.94** (14.71)	**77.38** (15.29)	**77.14** (14.76)	**74.51** (13.93)	**80.20** (13.01)

The bold values indicate the highest value within each column of data in the table.

**Table 2:** Comparison of average classification accuracy (%) and standard deviation (SD) for different methods (BCIC-IV-2a dataset).

	**Within-session**		**Cross-session**		**Two-session**
		**Session1 (SD)**	**Session2 (SD)**	**Case1 (SD)**	**Case2 (SD)**	**Session 1&2 (SD)**
CSP	57.75 (13.71)	60.60 (14.29)	54.01 (12.77)	54.07 (12.13)	57.15 (12.26)
FBCSP	73.57 (16.28)	72.46 (16.53)	65.59 (17.51)	65.79 (14.21)	75.01 (12.97)
MDM	62.96 (14.01)	59.49 (16.63)	-	50.74 (13.80)	-
TSM	68.71 (14.32)	63.32 (12.68)	-	49.72 (12.39)	-
SPDNet	65.91 (10.31)	61.16 (10.50)	-	55.67 (9.54)	-
Shallow ConvNet	71.83 (15.63)	72.64 (19.62)	74.61 (12.36)	68.96 (14.28)	78.83 (12.32)
EEGNet	69.26 (11.59)	66.93 (11.31)	61.65 (14.20)	60.31 (10.52)	70.67 (17.27)
FBCNet	**77.26 (14.82)**	**76.58 (13.09)**	-	72.71 (14.67)	-
Tensor-CSPNet	75.98 (14.26)	74.92 (14.63)	-	72.96 (14.98)	-
Proposed model	74.19 (10.60)	76.25 (12.67)	**75.85 (14.11)**	**75.08 (12.66)**	**81.04 (8.54)**

The bold values indicate the highest value within each column of data in the table.

In the KU dataset, our proposed model achieved the best performance in all scenarios, especially on the cross-session and two-session ones. Constrained by the limited data size of each subject, which only comprises 200 trials per session, achieving even slight improvements can be a challenge. In the within-session scenario, the proposed model achieved accuracy of 75.94% and 77.38% in session 1 and session 2 respectively. which are 0.99% and 1.46% higher than the best public model namely tensor-CSPNet. When utilizing twice the amount of data in the two-session scenario, the proposed model achieved a classification rate exceeding 80%, 7.46% higher than the Shallow ConvNet. In two cases of cross-session scenarios, as Figures 1B, C presented, both of them used the data from session 2 as the test and did not allow them to present in the training step. The difference was that case 1 used half of the data from session 2 to validate while case 2 did not use it. Considering the drift of data distributions caused by the different sessions conducted on different days, the results of cross-session are lower than the ones of within-session. The performances of most compared methods decrease including the proposed model. The existing high-performing models such as FBCNet and Tensor-CSPNet exhibited reduced performance to <70% while the proposed model only lost an average of 0.24% accuracy and still produced the best accuracy of 77.14% in case1. Given the BCI application that people often only used the data collected in one day to build the model without training or updating in the following day for saving patients' time, case 2 is more suitable in practical applications. The proposed model achieved 74.51%, a much higher accuracy than the benchmarks, which confirms the superiority of our proposed model on adaptability and robustness. The statistical test was also conducted to compare the performances of different models. We observed that the proposed model outperformed most baseline models (*p* < 0.001), FBCNet(*p* < 0.05) and Tensor-CSPNet (*p* < 0.05) in different scenarios.

In the BCIC-IV-2a dataset, FBCNet performed best in two within-session scenarios while the proposed model showed an accuracy decrease of 3.07% and 0.33% respectively in session 1 and session 2. However, in the two cross-session scenarios, our proposed model improved significantly which reached 75.84% in case1, 1.24% higher than the Shallow ConvNet and 75.08% in case2, 2.12% higher than the Tensor-CSPNet. The result in the two-session scenario also reached 81.04% which improved the accuracy by 2% compared to the Shallow ConvNet. The statistical test showed that the proposed model outperformed all baseline models (*p* < 0.05) in both of the cross-session and two-session scenarios.

We also checked the statistical significance between each scenario. The t-tests result of within-session 1 with within-session 2 was [*correlation* = 0.781, *p* < 0.001; *t*_(53)_ = −1.062, *p* = 0.293] and [*correlation* = 0.88, *p* < 0.01; *t*_(53)_ = −1.024, *p* = 0.336] in KU and BCIC-IV-2a dataset separately, which shows that there is consistency between different sessions for each subject, but the difference between two sessions is not statistically significant. In the KU dataset, the *t*-tests of within-session1 with case 1 and case 2 in the cross-session scenario were [*t*_(53)_ = −0.838, *p* = 0.406] and [*t*_(53)_ = 1.182, *p* = 0.242]. The t-tests in BCIC-IV-2a were [*t*_(53)_ = −0.627, *p* = 0.548] and [*t*_(53)_ = −0.475, *p* = 0.648]. Both results of the *t*-test did not have statistically significant differences. Hence, the data quality of an individual varies on different days. Building a model for each day is time-consuming and impractical, but employing a cross-session model may result in a decrease in classification accuracy, making it a challenging task. The t-tests results of within-session 1 with two-session and within-session 2 with two-session are [*correlation* = 0.882, *p* < 0.001; *t*_(53)_ = −4.514, *p* < 0.001] and [*correlation* = 0.901, *p* < 0.001; *t*_(53)_ = −3.102, *p* < 0.01] separately. Evidently, an increase in the volume of data contributes to the enhancement of model performance even though the sessions were collected on different days.

In summary, for the KU dataset, the proposed model outperforms other models, achieving up to 0.99% and 1.46% for the session 1 and 2 respectively in the within-session scenario, up to 7.49% and 8.19% for the case 1 and 2 respectively in the cross-session scenario and up to 7.46% for the two-session scenario. When testing on the BCIC-IV-2a dataset, the model can also improve the classification accuracy by 1.24% and 2.12% for the case 1 and 2 in the cross-session scenario and 2.21% for the two-session scenario, confirming the superiority of the proposed model in decoding MI-EEG information.

### 3.2. Ablation study

The purpose of an ablation study is to assess the impact of specific components on the overall performance of a model by removing them and analyzing their contribution. We conducted the ablation tests to evaluate the effectiveness of the transformer encoders, the hemisphere difference in the spatial block, and the T-Dense units in different scenarios. (1) The proposed model without transform encoders (w/o_Trans) removes both the local and global transformer encoders; (2) The proposed model without the hemisphere difference in the spatial block (w/o_Diff-hemi) removes the structures in Figure 3b which extract the spatial features from each hemisphere and calculate the difference. The previous models proposed in the literature which were shown to achieve good classification results in the KU EEG dataset such as EEGNet (Lawhern et al., 2018), Shallow ConvNet (Schirrmeister et al., 2017) and FBCNet (Mane et al., 2020b) focus on the spatial features from all channels in the motor region and ignore the available information that might be learned from the hemispheric differences. (3) The proposed model without T-dense units (w/o_T-dense) replaces the two T-dense units with common CNN layers. The results of the ablation study in different scenarios are shown in Table 3.

**Table 3:** Ablation study of the proposed method on the different modules.

	**Within-session**		**Cross-session**		**Two-session**
		**Session1 (SD)**	**Session2 (SD)**	**Case1 (SD)**	**Case2 (SD)**	**Session 1&2 (SD)**
**KU dataset**					
w/o_trans	75.81 (14.22)	68.01 (13..31)	75.02 (14.89)	66.42 (11.01)	72.88 (12.63)
w/o_diff-hemi	75.20 (14.92)	75.98 (15.59)	73.89 (16.01)	73.20 (13.98)	78.89 (13.60)
w/o_T-dense	67.88 (12.36)	68.53 (13.12)	68.54 (12.88)	66.74 (11.39)	73.61 (12.81)
Proposed model	**75.94 (14.71)**	**77.38 (15.29)**	**77.14 (14.76)**	**74.51 (13.93)**	**80.20 (13.01)**
**BCIC-IV-2a dataset**					
w/o_trans	**74.64 (11.21)**	76.07 (12.58)	73.14 (13.64)	73.64 (11.54)	**84.01(8.60)**
w/o_diff-hemi	72.56 (11.09)	73.95 (14.59)	70.67 (15.15)	73.72 (11.82)	78.91(10.08)
w/o_T-dense	63.31 (8.43)	65.74 (10.11)	68.82 (13.73)	63.12 (6.47)	77.21 (8.66)
Proposed model	74.19 (10.60)	**76.25 (12.67)**	**75.85 (14.11)**	**75.08 (12.66)**	81.04 (8.54)

The bold values indicate the highest value within each column of data in the table.

**Figure 5:**
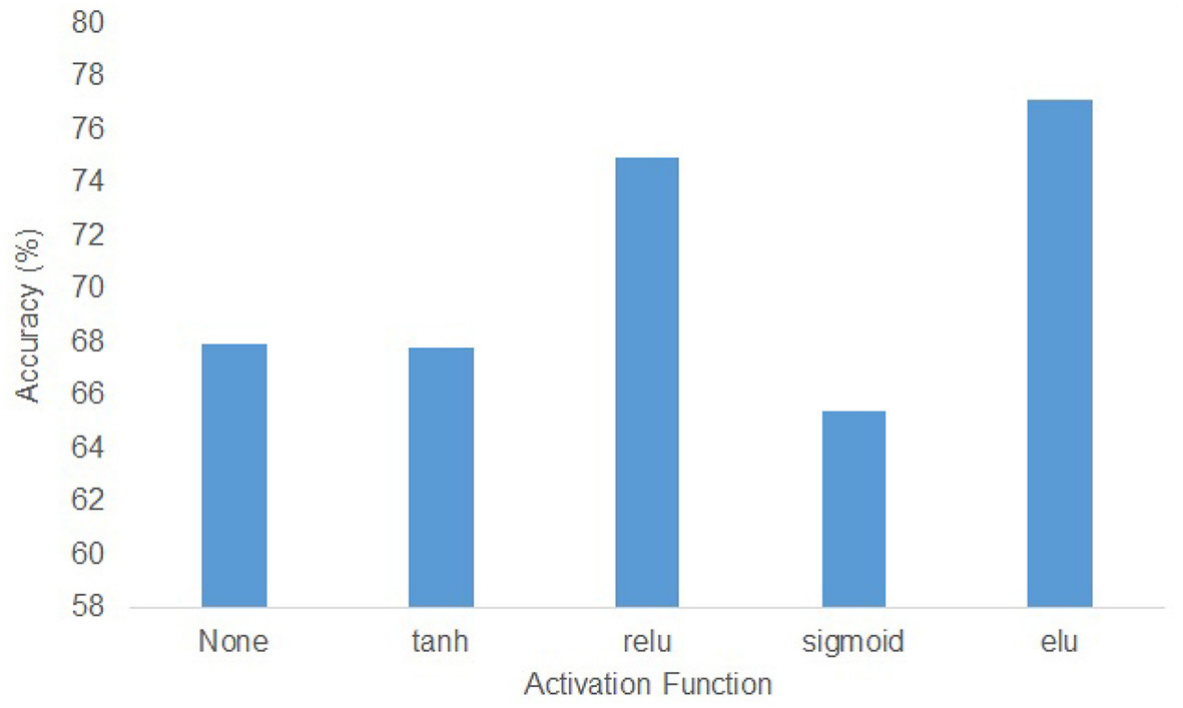
The effect of activation functions of all subjects in KU dataset.

In the KU dataset, the T-test results indicated that there is no statistical significance (*p* > 0.05) to show the w/o_trans brings a negative impact on the classification accuracy in session 1 of the within-session scenario. Apart from this, the absence of any specific components will make the accuracy drop(*p* < 0.05). Especially for session 2 in the within-session scenario, without the transformer encoders, the classification result decreased by 9.37%. In the BCIC-IV-2a dataset, although in within-session1 and two-session scenarios, the model without transformer encoders performed better, other cases still show the importance of the different modules. The statistical analysis showed that w/o_trans decreased the classification accuracy in the cross-session scenario (*p* < 0.05). The w/o_Diff-hemi had significance in within-session and cross-session scenarios (*p* < 0.05) while the T-test result in the two-session scenario was *p* = 0.127. The w/o_T-dense had statistical significance in all scenarios (*p* < 0.01). It is clear that the T-dense has significant contributions to the classification accuracy improvement, as it produces a comprehensive set of temporal-spatial features. Without this module, using a simple temporal block and spatial block is unable to capture sufficient and subtle useful features embedded in the highly corrupted and diffused EEG raw data. We also tested the selection of the activation functions on cross-session case 1 from the KU dataset (Figure 5). The ELU function performed best with the highest accuracy.

### 3.3. Complexity

Table 4 shows the model complexity based on the number of trainable parameters. The results show that there is no decisive relationship between the complexity of a model and its performance. Deep ConvNet has the most parameters because of more CNN layers used in the structure. However, regardless of the scenarios, the Deep ConvNet performs badly even worse than the traditional approach FBCSP. Among these compared models, the EEGNet only has no more than 2k trainable parameters because the depthwise separable convolution layer is employed to reduce the dimensions. However, EEGNet performs much better than the Deep ConNet in each scenario. The Tensor-CSPNet divides the raw signals into several frequency bands to learn subtle features within different frequency bands, thus encompassing the spectral differences among different subjects. This approach adds additional computational parameters but the model performance is the best as demonstrated in the previous studies. The proposed model includes 12K of trainable parameters that are only half of the Tensor-CSPNet but has better classification results, which demonstrated its efficacy and effectiveness.

**Table 4:** Model complexity based on the number of trainable parameters.

**Models**	**Parameters**
Shallow ConvNet	42,884
Deep ConvNet	282,004
EEGNet	1,876
Tensor-CSPNet	232,360
Proposed Model	118,337

### 3.4. Feature visualization

**Figure 6:**
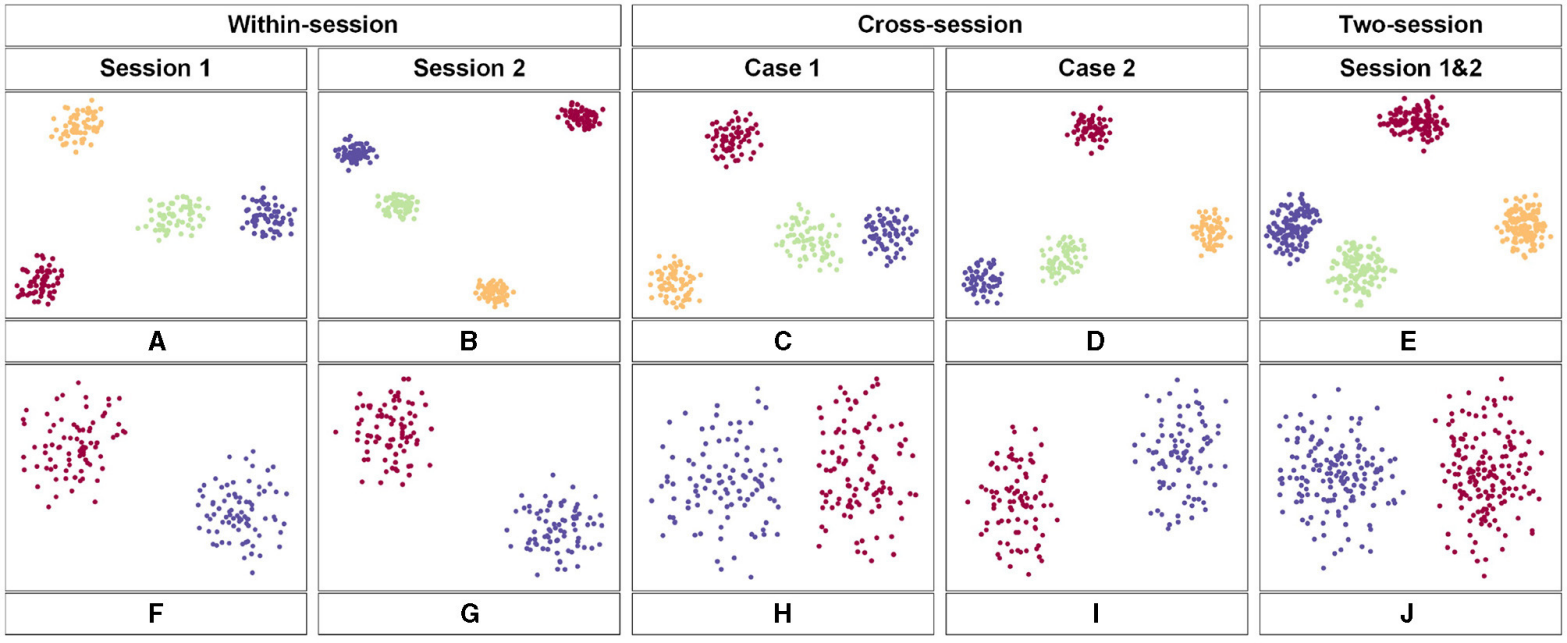
The feature map obtained by the proposed model in 2-D embedding based on t-SNE. **(A–E)** is the distribution of the extracted features of the third subject from the BCIC-IV-2a dataset. **(F–J)** show the distribution of extracted features of the third subject from the KU dataset.

The t-distributed Stochastic Neighbor Embedding (t-SNE) approach was employed to visualize the feature distribution after the last fully connected layer of the proposed model. Figure 6 shows the comparison of the visualization based on the different scenarios. We used the data from subject 3 in the two datasets respectively. Figures 6A–E belongs to the BCIC-IV-2a while Figures 6F–J belongs to the KU dataset. Each color represents one label of MI-EEG tasks. According to the t-SNE result, the proposed model showed a great ability to classify EEG signals. In comparison to within-session, the feature distribution in cross-session and two-session scenarios appears to be more dispersed. However, there are still clear distinctions that can be observed, further showing the superior performance of the proposed model.

## 4. Discussion

In this work, we proposed a local and global convolutional transformer-based model for MI-EEG classification. The transformer encoder with the self-attention mechanism is widely applied to the computer version and natural language processing. Compared with the CNN limited by the size of its filter, the transformer can capture all samples simultaneously, which is suitable to extract global features. Meanwhile, the calculation step of the self-attention mechanism focuses on finding the relationship of different features while CNN extracts common mode from features. Once the CNN-based model is trained, the weights in the filters are fixed. However, in a transformer encoder, the weights depend on the inputs, so they are dynamically changed according to the data. Previous studies have shown that the EEG, as intricate time series, varies from subject to subject which makes the transformer a suitable approach for processing EEG signals. Due to the distinct characteristics of CNN and transformer, combining and complementing each other makes for exploring more useful features of EEG signals and ensuring the robustness of the model.

In the proposed model, we employed two strategies for the transformer, specifically the local and the global modes. When extracting temporal features from raw EEG signals, such a long time series will significantly increase the cost of model computation and lead to severe overfitting problems. Using the local transformer encoder can limit the size of the filter like the learning mode of a CNN layer. Although this will cause the transformer to lose the chance of obtaining global features of long sequences at once, it can still leverage the advantage of dynamically extracting learning feature relationships, complementing the CNN. When the features are sent into the T-Dense block, the transformer encoder employs the global mode because the time series has been processed with the pooling layers. Meanwhile, the feed-forward fashion connecting each layer to every other layer in the CNN and transformer branch encourages feature reuse and information flow which improve the model performance. In the spatial block, compared with previous models the proposed model used the depthwise CNN layer to extract spatial features not only from all channels like ConvNet (Schirrmeister et al., 2017), EEGNet (Lawhern et al., 2018) and FBCNet (Mane et al., 2020b) which performed well in the KU and BCIC-IV-2a dataset but also from the difference of two hemispheres. The result of the ablation study has shown the efficiency of this module. After extracting features from the hemisphere differences, the proposed model got higher classification results in all scenarios, especially in the cross-session cases.

To better validate the superiority of the proposed model, we designed three scenarios including within-session, cross-session, and two-session in two famous public datasets. From Tables 1, 2, the results show that our proposed model achieved the highest classification result in the different scenarios. Compared with the other two scenarios, the cross-session scenario is closer to the real application which limits the model performance because of the number of data and the drift of statistical distributions. However, our proposed model still performed well and was less than only 3% than within-session results which further shows the good robustness and adaptability. Previous models based on the transformer for MI classification use the CNN layers (Ma et al., 2022; Xie et al., 2022; Song et al., 2023) to extract temporal features while the transformer is used to refine features. While the proposed model adopted the local mode of the transformer to complement the functionality of CNN in time-series data analysis, rather than simply placing the transformer behind the CNN layer. Meanwhile, during the feature refinement stage, the proposed model not only employed the attention mechanism in the transformer but also combined with the DenseNet to improve the flow and reuse of information. Further, the spatial features learned from the difference of the hemispheres were also taken into consideration.

Although our proposed model has shown superior performance than previous methods, there is still room for improvement. First, the proposed model only adopts the transformer encoder on the time series but ignores the possible spatial features extracted based on the self-attention mechanism. The main reason we did not add this module is that the overall length of the sequence is quite long after obtaining temporal features. If we use a transformer to learn the correlation between each channel's features and replace the deepwise convolutions, it will cause severe overfitting problems. Meanwhile, although the complexity based on the trainable parameters of our model is not high, the computation time is still large because the local transformer slid to process inputs like CNNs which is time-consuming. Also compared with other transformer-based models, the selection of the position encoding methods is not considered in the proposed model. Thus, a future work will investigate a more efficient model structure. Secondly, self-attention can help reallocate the weights that present the importance of each feature. In future works, investigations and visualization of these weights to expand the interpretability and mechanism corresponding with the nerve disease are desirable to study.

## 5. Conclusion

In this article, we have presented a novel and effective approach for MI-EEG classification using a local and global convolutional transformer-based model. The proposed model has been validated on the three scenarios and two public datasets. The combination of CNN filters and transformer encoders with local and global structures has the advantage of extracting a comprehensive set of useful features from EEG signals. In the spatial module, we also consider the possible information from the differences between the hemispheres which helps improve the robustness of the model. Our results showed that the proposed model outperformed the state-of-the-art methods for MI-EEG classification on the KU dataset, achieving up to 0.99% and 1.46% for the session 1 and 2 respectively in the within-session scenario, up to 7.49% and 8.19% for the case 1 and 2 respectively in the cross-session scenario and up to 7.46% for the two-session scenario. For the BCIC-IV-2a dataset, the model can also improve the classification accuracy by 1.24% and 2.12% for the case 1 and 2 in the cross-session scenario and 2.21% for the two-session scenario.

## Data availability statement

Publicly available datasets were analyzed in this study. This data can be found here: https://academic.oup.com/gigascience/article/8/5/giz002/5304369.

## Ethics statement

The studies involving humans were approved by Korea University Institutional Review Board (1040548-KUIRB-16-159-A-2). The studies were conducted in accordance with the local legislation and institutional requirements. Written informed consent for participation was not required from the participants or the participants' legal guardians/next of kin in accordance with the national legislation and institutional requirements.

## Author contributions

JZ: conceptualization, investigation, methodology, software, and writing—original draft. KL: conceptualization, supervision, and modification. BY: conceptualization and modification. XH: investigation and methodology. All authors contributed to the article and approved the submitted version.
